# Assessment of lutein, zeaxanthin and *meso*-zeaxanthin concentrations in dietary supplements by chiral high-performance liquid chromatography

**DOI:** 10.1007/s00217-015-2569-9

**Published:** 2015-10-07

**Authors:** Alfonso Prado-Cabrero, Stephen Beatty, Alan Howard, Jim Stack, Philipp Bettin, John M. Nolan

**Affiliations:** Macular Pigment Research Group, Vision Research Centre, Department of Chemical and Life Sciences, Waterford Institute of Technology, Carriganore House, Waterford, Ireland; Downing College, The Howard Foundation, University of Cambridge, Cambridge, UK; Institute of Pharmaceutical Biology, University of Greifswald, Greifswald, Germany

**Keywords:** *meso*-zeaxanthin, Lutein, Zeaxanthin, Macula, Chiral HPLC–DAD

## Abstract

We investigated the concordance between actual and declared content of the three macular carotenoids in commercially available supplements aimed at eye health. Three batches of nine products were tested for content of lutein (L), zeaxanthin (Z) and *meso*-zeaxanthin (MZ) by chiral HPLC–DAD. In every product tested, actual L concentration was close to target, but Z concentration varied greatly (47–248 % of declared concentration), and the L:Z ratio within some supplements was adversely affected in consequence. In six of seven products not declaring MZ, we found this carotenoid, and four of them, using the same L source, contained a concentration of MZ that correlated positively and significantly with measured concentrations of L (*r*^2^ = 0.86; *P* < 0.001). More transparency is needed in terms of concordance between actual and declared concentrations of Z in commercially available formulations, and MZ should be declared in those formulations where it is present.

## Introduction

**Fig. 1:**
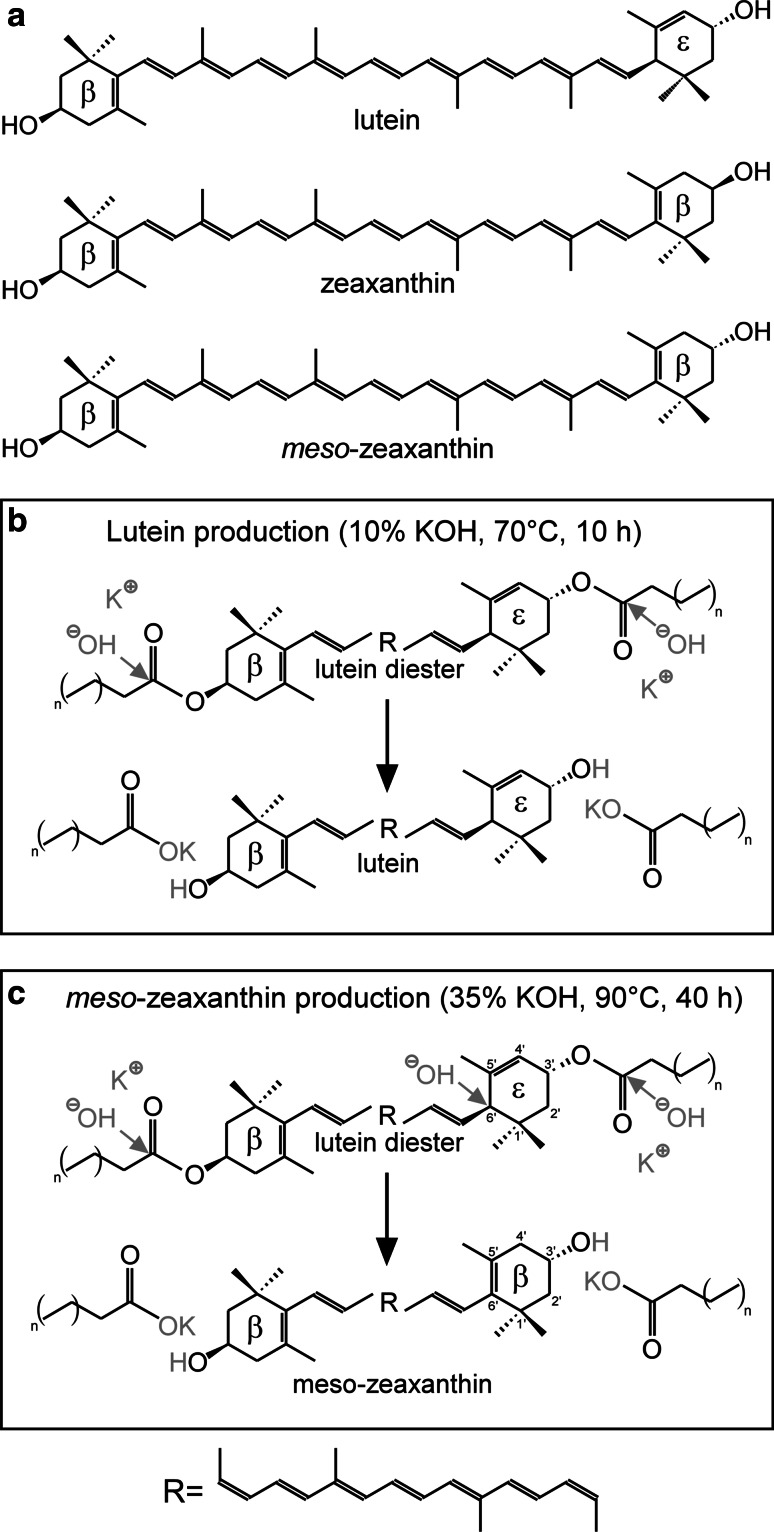
Structure of the three macular carotenoids and processes performed to obtain lutein and *meso*-zeaxanthin from marigold. **a** Structure of lutein [(3R,3’R,6’R)-β,ε-Carotene-3,3’-diol], zeaxanthin [(3R,3’R)-β,β-Carotene-3,3’-diol] and *meso*-zeaxanthin [(3R,3’S)-β,β-Carotene-3,3’-diol]. **b** Lutein production process from marigold petals with saponification conditions described previously [33]. **c** *meso*-zeaxanthin production process from marigold with saponification conditions as described previously [34]. Lutein diester is attacked by one hydroxyl group (OH^−^) at carbon C6´ to trigger the conversion of the ε-ring to a β-ring, as suggested by Andrewes et al. [23]. *n* stands for 6, 7 or 8 repetitions of the two carbons included in the *brackets*, indicating that the fatty acid can be miristic, palmitic or stearic acid

The central region of the retina, known as the macula, is responsible for central and colour vision [1] and is yellow in colour (hence *macula lutea*) due to the accumulation of the carotenoids lutein (L), zeaxanthin (Z) and *meso*-zeaxanthin (MZ) (Fig. 1a) [2], where these compounds are collectively referred to as macular pigment (MP). The short-wavelength (blue) light-filtering properties of MP are important for optimal vision (because of consequential attenuation of chromatic aberration and the adverse impact of light scatter) [3, 4] and also confer photoprotection to the central retina because short-wavelength (blue) light is the most injurious of visible wavelengths. Further, the macular carotenoids actively quench damaging free radicals, and this antioxidant effect is maximized when all three carotenoids are present [5].

L and Z are consumed in a typical diet containing fruits and leafy green vegetables, whereas MZ has not been detected in collard greens [6]. It has been reported that in a typical Western diet, intake of L is between 1.0 and 3.6 mg/day and intake of Z is circa 0.1 mg/day [7, 8]. However, MZ has been detected in liver of frog and quail [9] and more recently in trout flesh [10]. In the retina, there is evidence that macular MZ is derived (at least in part) from retinal L [11, 12], and given the scarcity of dietary sources of MZ, it is likely that most of retinal MZ is formed from L, but further work is needed to confirm this hypothesis [13–15].

In the vast majority of subjects, MP can be augmented following supplementation with MP’s constituent carotenoids [16], suggesting less-than-saturation levels in a substantial proportion of the population. Of note, commercially available supplements used for eye health declare concentrations between 2 and 22 mg of total carotenoid. However, the ratio of the respective macular carotenoids used in these formulations differs between products. There are a large number of trials reporting on the impact of supplementation with at least two of the three macular carotenoids (typically 10 mg/day of L and 2 mg/day of Z), and there is strong evidence that supplemental L and Z benefit patients with non-advanced age-related macular degeneration (AMD), both in terms of disease progression and in terms of visual loss [17, 18]. However, a formulation containing all three macular carotenoids (typically 10 mg/day of L, 2 mg/day of Z and 10 mg/day of MZ) does appear to offer some advantages in terms of augmentation of macular pigment across its spatial profile, and in terms of improvements in visual performance in diseased [19, 20] and non-diseased eyes [21]. Further, given the relatively recent commercial availability of MZ for inclusion in formulations, there is need to assess its inclusion (whether overt, covert or inadvertent) in commercially available formulations containing the macular carotenoids.

In order to generate a dietary supplement, manufacturers may obtain L and MZ from a number of source companies, which in turn obtain them from the petals of the marigold flower (*Tagetes erecta*). In order to extract L, the marigold petals are submitted to a saponification process that liberates L of linked fatty acids (Fig. 1b). In order to produce MZ, the saponification process is modified to isomerize L to MZ through a double-bond migration that turns the ε-ring into a β-ring [22, 23] (Fig. 1c). In order to produce Z, several sources can be used, including hybrid varieties of marigold containing high concentrations of Z [24], paprika (*Capsicum annuum*) [25], wolfberry (*Lycium barbarum*) [26], *Flavobacterium multivorum* [27] and the fruit Sastra (*Garcinia intermedia*) as a newly identified rich source of this carotenoid [28]. Moreover, it has recently been suggested that some companies have used MZ as a substitute for Z, perhaps prompted by the commercial observation that MZ is cheaper to source than Z, given that these carotenoids cannot be distinguished with standard HPLC techniques [29].

We have reported the presence of undeclared MZ in commercially available eye care formulations in the past [30], and that observation prompted us to conduct the current study on concordance between declared and actual concentrations of the macular carotenoids in commercially available formulations and to investigate the implications of our observations for possible sources of undeclared MZ in such supplements. We believe that this study is important, primarily because it will help facilitate the investigation of agreement between declared and actual concentrations of the carotenoids present in commercially available eye supplements. The results from this study will also have important implications for designing and interpreting clinical studies where supplementation with the macular carotenoids is under investigation. Finally, the information emanating from this study will inform eye care specialists on their choice of a commercially available formulation for their patients.

## Materials and methods

### Supplements analysed

In this study, we measured the carotenoid content of nine commercially available formulations containing the macular carotenoids (Table 1). Three batches, different in terms of expiry date, were sourced for each product and analysed (in triplicate) in order to investigate possible variability between batches. All formulations were presented as soft gel capsules, with the carotenoids suspended in fish oil or vegetable oil. Of note, some formulations also contained multivitamins and/or co-antioxidants.

**Table 1:** Concentration of lutein (L), zeaxanthin (Z) and *meso*-zeaxanthin (MZ) in eye care supplements

Supplement name, manufacturer, carotenoid supplier	Batch number	Months expiry	Carotenoids (mg/capsule)			% Achieved (95 % confidence)
			Declared		Measured	
**Preservision AREDS2** ^**®**b^	2710E0566	16	**L**	5	6.05 ± 0.27	121 (117–125)
Bausch + Lomb^®^└	2936E0566	16	**Z**	1	1.63 ± 0.11	163 (155–172)
Floraglo^®^ Lutein	2939E0566A	16	**MZ**	*	nd	–
**VitaluxPlus** ^**®a**^	E02010	13	**L**	10	11.12 ± 0.52	111 (107–115)
Alcon^®^	E05507	16	**Z**	2	1.03 ± 0.07	52 (49–54)
Floraglo^®^ Lutein	E03745	15	**MZ**	*	0.10 ± 0.01	–
**Nutrof** ^**®**^ **omega** ^a^	V067	10	**L**	10	9.54 ± 0.67	95 (90–101)
Spectrum Thea^®^	V070	12	**Z**	2	1.30 ± 0.27	47 (44–50)
Floraglo^®^ Lutein	V063	8	**MZ**	*	0.94 ± 0.08	–
**Ultra Lutein** ^**®a,c**^	1266679	17	**L**	20	20.78 ± 0.73	104 (101–107)
Nature’s Plus^®^	1263243	14	**Z**	0.86	2.13 ± 0.09	248 (240–256)
Floraglo^®^	1268878	17	**MZ**	*	0.18 ± 0.03	–
**Eyepromise Restore** ^**®**b^	C1401047	28	**L**	4	4.83 ± 0.14	121 (118–123)
Zeavision^®^	H13059	22	**Z**	8	1.28 ± 0.07^#^	16 (15–17)
Floraglo^®^ Lutein, Zeagold^®^	B14045	28	**MZ**	*	0.04 ± 0.01	–
**CentroVision** ^**®**^ **L** **forte** ^a^	5054	15	**L**	14	13.91 ± 0.45	99 (97–102)
OmniVision GmbH^®^	4581	10	**Z**	1.04	1.68 ± 0.08	161 (156–167)
Floraglo^®^ Lutein	8180	10	**MZ**	*	0.11 ± 0.01	–
**MacuHealth with LMZ3** ^**® d**^	110614	29	**L**	10	10.89 ± 1.34	109 (99–119)
Macuhealth LLC^®^	160314	26	**Z**	2	2.19 ± 0.49	109 (90–128)
IOSA^®^	330913	20	**MZ**	10	12.15 ± 2.14	122 (105–138)
**MacuShield** ^**® d**^	116215	11	**L**	10	12.11 ± 0.91	121 (114–128)
Macuvision Europe^®^	118860	21	**Z**	2	2.51 ± 0.25	126 (116–135)
IOSA^®^	120480	26	**MZ**	10	12.70 ± 0.74	127 (121–133)
**Ocuvite** ^**®**^ **L Plus** ^**a**^	D09592	10	**L**	5	5.53 ± 0.26	111 (107–115)
Bausch + Lomb^®^	D09588	10	**Z**	1	0.60 ± 0.03	60 (57–62)
Unknown	D09591	10	**MZ**	*	0.79 ± 0.03	–

Carotenoid amounts are provided in mg/capsule ± standard deviation, and as percentage of declared concentration achieved, including 95 % confidence interval

^a^Sourced in pharmacy

^b^Sourced online

^c^Sourced in health food store

^d^Sourced by the manufacturer

* Carotenoid not declared in product label, nd, carotenoid not detected

– Non-applicable

^#^ Quantification not including esterified Z

We determined that MacuHealth with LMZ3^®^ and MacuShield^®^ use L, Z and MZ from IOSA (Industrial Orgánica S.A., Monterrey, Nuevo León, Mexico), whereas (where known) the rest of the formulations use the product FloraGLO^®^ Lutein (Kemin Industries, Inc., Des Moines, IA, USA). Only Ocuvite^®^ L Plus did not specify the source of carotenoids used on the product label. Eyepromise Restore^®^ was the only supplement to specify a source of Z (Zeagold^®^, Calsek Inc., Kalamazoo, MI, USA).

### Carotenoid standards and solvents

L standard [(3R,3’R,6’R)-β,ε-Carotene-3,3’-diol] and Z standard (racemic mixture of the three Z enantiomers: (3R,3’R)-β,β-Carotene-3,3’-diol), (3S,3’S)-β,β-Carotene-3,3’-diol and (3R,3’S)-β,β-Carotene-3,3’-diol) were supplied by CaroteNature GmbH (Ostermundigen, Switzerland). The Standard Reference Material (SRM) 968e (Fat-Soluble Vitamins, Carotenoids, and Cholesterol in Human Serum) was obtained from NIST (National Institute for Standards and Technology, Gaithersburg, MD, USA). The solvents THF (tetrahydrofuran), methanol, MTBE (methyl *tert*-butyl ether), hexane and isopropanol, all HPLC grade, were purchased from Sigma-Aldrich (Vale Road, Arklow, Wicklow, Ireland) or Thermo Fisher Scientific (Blanchardstown Corp Pk 2, Ballycoolin, Dublin, Ireland). BHT (butylated hydroxytoluene) was purchased from Sigma-Aldrich.

### Statistical methods

The statistical software package SPSS 20 was used for analysis. The focus of the study was on agreement between declared and actual carotenoid content for each supplement and on variation in this agreement between batches of supplements. The study also investigated, for each supplement, achievement of target ratios for the respective carotenoids. As the formulations varied widely in terms of declared values (e.g. from 4 mg up to 20 mg for L), analysis was largely confined to data representing percentage agreement with declared values and achievement (at a minimum) of declared content of each respective carotenoid. Confidence intervals for mean percentage agreement for each formulation were generated in order to identify those supplements which failed to achieve close to 100 % agreement with declared carotenoid content. Between-batch variation, in terms of percentage agreement, was investigated using analysis of variance, separately for each supplement, with batch as a random factor. Achievement of target carotenoid ratios, for each supplement, was investigated by generation of confidence intervals for these ratios. The 5 % level of significance was used in all analyses, without adjustment for multiple tests, e.g. 95 % confidence intervals were used throughout. Confidence intervals were constructed treating the nine sample values for each supplement as independent, i.e. ignoring inter-batch variation, which was generally small. Data from different batches were also combined for regression analysis of the relationship between L concentration and undeclared MZ concentration in certain formulations.

### HPLC analysis

L, Z and MZ were separated and quantified on an Agilent Technologies (Palo Alto, CA) 1260 Series HPLC system equipped with a Diode Array Detector (DAD, G1315C), binary pump, degasser, thermostatically controlled column compartment, thermostatically controlled high-performance autosampler (G1367E) and thermostatically controlled analytical fraction collector. For system control and data processing, the software ChemStation for LC3D systems Rev. B.04.03-SP1 [87] (Agilent Technologies) was used. The standard injection volume was 10 µL.

System 1 (for carotenoid quantification) was performed using a Daicel Chiralpak AD-H column, composed of amylose tris (3,5-dimethylphenylcarbamate) coated on 5 μm silica gel (250 × 4.6 mm i.d.; Chiral Technologies Europe, Cedex, France). The column was protected with a guard column containing a guard cartridge with the same chemistry of the column (10 × 4 mm i.d. 5 µm). Isocratic elution was performed with hexane and isopropanol (95:5, v/v) and a flow rate of 1 mL min^−1^. The column temperature was set at 25 °C.

System 2 (for carotenoid ester analysis) was performed using a C30-reversed phase column (250 × 4.6 mm i.d., 3 µm; YMC Europe, Dinslaken, Germany) with a guard column containing a guard cartridge with the same chemistry of the column (10 × 4 mm i.d., 3 µm). The flow rate was 1 mL min^−1^ with a linear gradient from 100 % A (methanol: methyl *tert*-butyl ether:water (30:10:1, v/v/v)) to 20 % B (methanol: methyl *tert*-butyl ether (1:1, v/v)) within 10 min, then to 100 % B within 1 min, maintaining this condition for another 24 min. The solvents were returned to the starting conditions within 1 min, and the column temperature was set at 25 °C.

### Sample preparation

Sample extraction and preparation were performed under protective amber light provided by LED lamps installed in our laboratory (Philips BCP473 36xLED-HB/AM 100-277 V) in order to prevent carotenoid isomerization. The antioxidant BHT was added to the extraction solvents to prevent carotenoid degradation. For each supplement, a single capsule was selected at random and placed in a 50-mL polypropylene tube containing 10 mL of THF with 0.1 % BHT. The capsule was broken with a blade, allowing the content to flow into the THF. The blade was washed with 10 mL of THF to reach a final volume of 20 mL. Each tube was vortexed for 10 s, sonicated at 24 °C for 2 min and vortexed again for 10 s, in order to efficiently separate the capsule contents from the shell. The tubes were centrifuged at 4700 rpm at 25 °C without brake to avoid resuspension of the pellet. Different dilutions of each tube were then prepared for the following purposes: detection of MZ, dilution 1:10; quantification of Z and MZ, dilution 1:100; quantification of L, dilution 1:600. 0.2 mL of each dilution prepared were dried in a centrifugal vacuum concentrator (GeneVac MiVac Duo Concentrator, Ipswich, England) and resuspended in 2 mL of HPLC mobile phase.

**Fig. 2:**
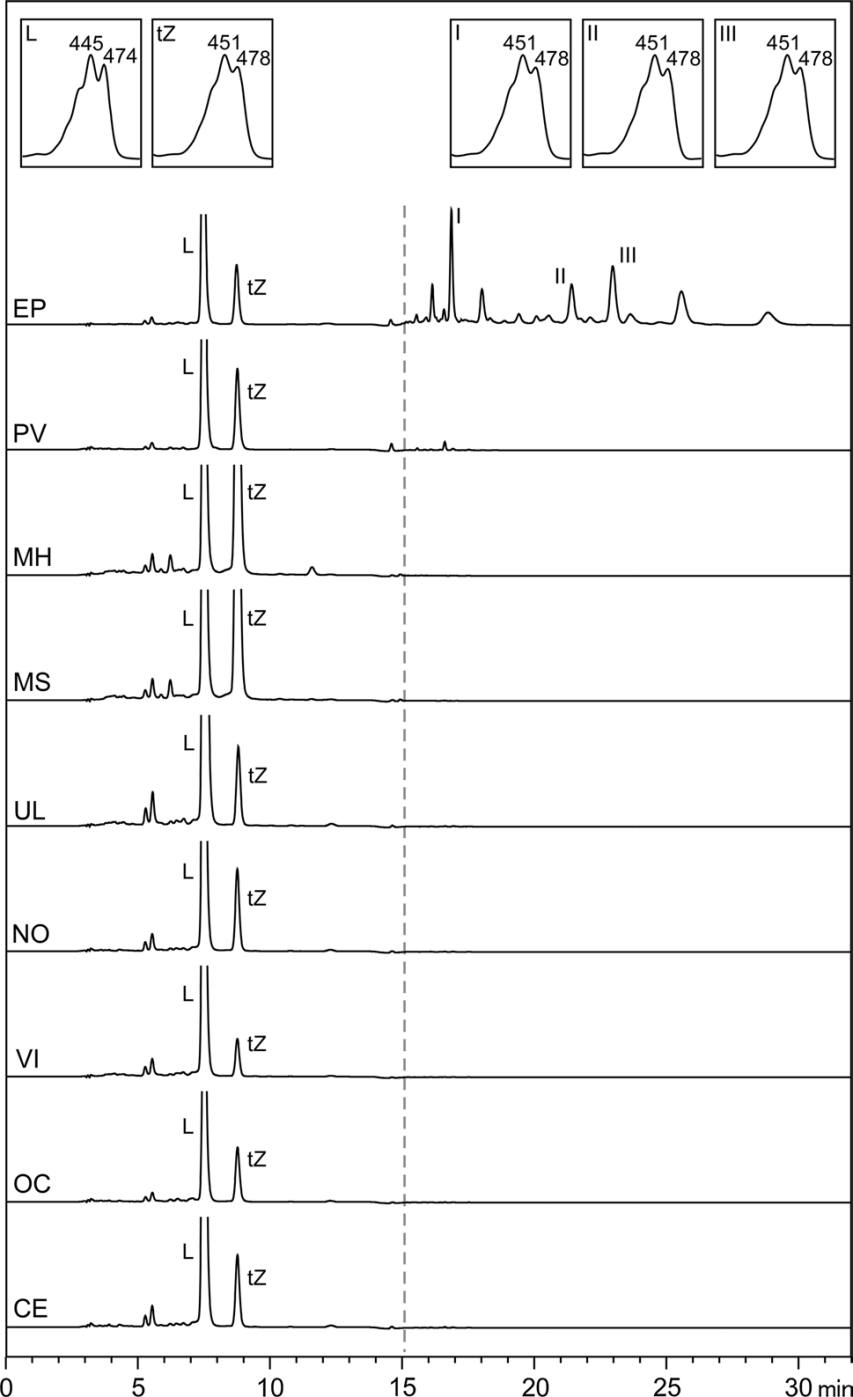
Detection of free carotenoids and carotenoid esters in the supplements using HPLC–DAD with a C30 column. Unesterified lutein (L), unesterified total zeaxanthin (tZ) and esterified zeaxanthin (peaks I–III as example). *EP* Eyepromise Restore^®^; *PV* Preservision AREDS2^®^; *MH* MacuHealth with LMZ3^®^; *MS* Macushield^®^; *UL* Ultra Lutein^®^; *NO* Nutrof^®^omega; *VI* Vitalux Plus^®^; *OC* Ocuvite^®^ L Plus; *CE* CentroVision^®^ L Forte

We analysed the supplements using HPLC system 2 and verified the presence of non-esterified carotenoids (Fig. 2), thereby precluding the need for saponification. However, for one supplement (EyePromise Restore) we identified esterified Z in the formulation, and, given that esterified Z was not quantified as part of this study, this supplement was not included in analyses regarding this carotenoid.

In order to validate the method, we performed a recovery assay of L using the product Ultra Lutein^®^ and L standard as described previously [31]. We carried out the assay in duplicate, and the extraction efficiency was 95.2 ± 2.3 %.

### Calibration

We confirmed the accuracy of our HPLC system using the Standard Reference Material 968e level 2 (NIST) in HPLC system 2. L measured was 0.098 µg mL^−1^, which is within the limits of the certified value reported by NIST for this carotenoid (0.097 ± 0.007 µg mL^−1^).

Quantification was achieved by constructing two standard curves, one for L and one for Z and MZ, using a UV–Vis spectrophotometer UVmini-1240 (Shimadsu) and HPLC system 1, with the DAD detector set to 450 nm. For the standard L curve, L was previously purified in our laboratory up to 94 % purity (based on peak area). Seven concentrations were measured in triplicate within a linear range 0.4–3.6 mg L^−1^, and the molar extinction coefficient applied was 147.3 × 10^3^ L mol^−1^ cm^−1^ in hexane [32]. The resulting regression line was given by the formula *y* = 10.70*x* − 15.22 (*r*^2^ = 0.998), where *x* is L concentration and *y* is the peak area. For the Z and MZ standard curve, the MZ enantiomeric mix from Carotenature was used (95 % purity based in peak area, system 2). Identical spectral characteristics were assumed for the three enantiomers present in the standard; therefore, a Z molar extinction coefficient was applied, 141.1 × 10^3^ L mol^−1^ cm^−1^ in hexane (Davies, 1976). Seven concentrations within a linear range 0.15–1.4 mg L^−1^ were used, and the resulting regression line was given by the formula *y* = 10.05*x* − 5.91 (*r*^2^ = 0.997), where *x* is Z concentration and *y* is the peak area.

We established the limit of quantification (LOQ) of our HPLC system for L and Z assessing the lowest concentration of each carotenoid quantifiable with a relative standard deviation (RSD) lower than 5 % (11 sample injections per carotenoid). LOQ was 0.62 ng for L (RSD = 3.1 %) and 0.15 ng for Z (RSD = 4.7 %).

### Inter-batch variability

In order to investigate the representativeness of our findings and the homogeneity of different batches, we measured the macular carotenoid content of three different batches of each formulation and assessed inter-batch homogeneity, of total carotenoid content measured, by analysis of variance with batch as a random factor. For one product, MacuHealth with LMZ3^®^, this analysis suggested statistically significant inter-batch differences in terms of percentage of total macular carotenoid content (*P* = 0.011); for this formulation, the amount of total carotenoids ranged from an average of 97.5 % of declared content for one batch, up to 133.2 % for another batch. None of the other formulations exhibited statistically significant inter-batch variability in terms of total carotenoid content. These results suggest that, for almost all formulations, inter-batch variability was not responsible for the findings reported herein, and, further, that the generation of confidence intervals could be legitimately based on treating the data as nine independent observations for each formulation. Moreover, although MacuHealth with LMZ3^®^ did exhibit inter-batch differences, it should be noted that even in the poorest batch of this formulation, the 95 % confidence interval (67–123 %) comfortably included 100 % of total declared carotenoid content.

## Results

### Concordance of each carotenoid to declared label concentrations

Table 1 presents the concentrations (mean ± SD of nine samples) of L and Z calculated for each formulation tested, along with per cent of target achieved and 95 % confidence intervals.

### Declared and measured ratios of L:Z and L:MZ

Table 2 presents the ratio of L:Z and L:MZ for each formulation tested, along with 95 % confidence intervals.

**Table 2:** Carotenoid ratios in eye care supplements

Supplement	L:Z			L:MZ		
	Declared	Measured	Conf. interval	Declared	Measured	Conf. interval
EyePromise restore^®^	0.5	3.8*	–	–	113.0	–
Ultra Lutein^®^	23.3	9.8	(9.4, 10)	–	116.5	–
CentroVision^®^ L forte	13.5	8.3	(8.0, 8.5)	–	132.7	–
VitaluxPlus^®^	5	10.8	(10.4, 11.3)	–	111.4	–
Ocuvite^®^ L Plus	5	9.3	(8.9, 9.6)	–	7.0	–
Nutrof^®^omega	5	10.1	(9.7, 10.6)	–	7.4	–
Preservision AREDS2^®^	5	3.7	(3.6, 3.8)	–	–	–
MacuHealth with LMZ3^®^	5	5.0	(4.6, 5.6)	1	0.91	(0.81, 0.99)
MacuShield^®^	5	4.8	(4.7, 5.0)	1	0.95	(0.92, 0.996)

Declared lutein:zeaxanthin ratios (L:Z) and lutein:*meso*-zeaxanthin ratios (L:MZ) were obtained by dividing the amount of declared L by the amount of declared Z or MZ, respectively. Measured ratios were obtained from the amounts of quantified L, Z and MZ. 95 % confidence intervals are displayed

* Bias in the measured L:Z ratio (esterified Z not quantified for this supplement)

– Non-applicable, MZ is not declared by the supplement

### Detection of undeclared MZ and its presence in FloraGLO^®^ Lutein

**Fig. 3:**
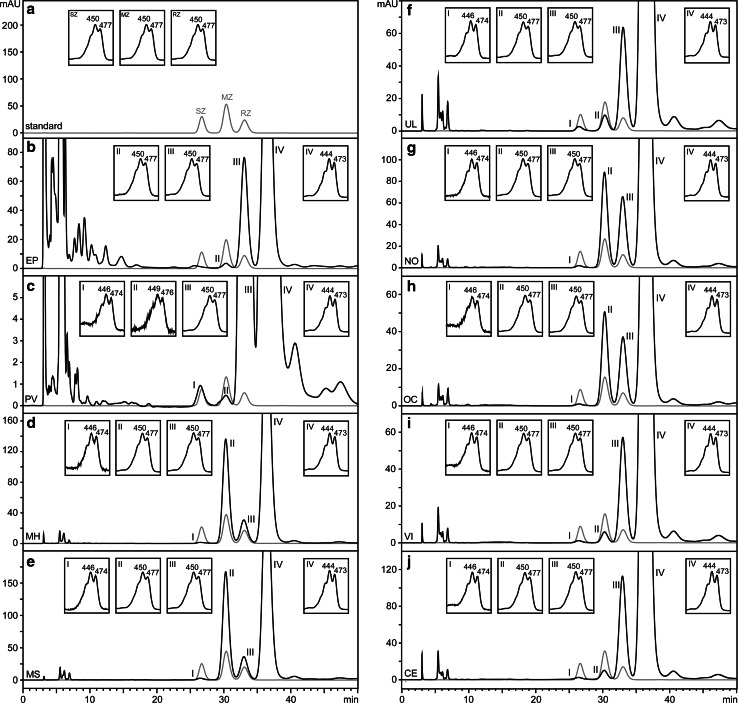
Detection of MZ in the supplements by chiral HPLC–DAD. A commercial standard containing a zeaxanthin racemic mixture, including (3S,3’S)-zeaxanthin (SZ), (3R,3’S)-zeaxanthin (MZ) and (3R,3’R)-zeaxanthin (RZ) was run, and the resulting chromatogram was overlaid (represented in *grey*) to the chromatograms of the supplements. Peak I, tentatively classified as oxo-lutein; peak II, (3R,3’S)-zeaxanthin (MZ); peak III, (3R,3’R)-zeaxanthin (RZ); peak IV, lutein (L). The *insets* represent the spectra of the peaks selected, specifying the maxima absorption wavelength (wavelength range 350–550 nm). *EP* Eyepromise Restore^®^, *PV* Preservision AREDS2^®^, *MH* MacuHealth with LMZ3^®^, *MS* Macushield^®^, *UL* Ultra Lutein^®^, *NO* Nutrof^®^omega, *OC* Ocuvite^®^ L Plus, *VI* Vitalux Plus^®^, *CE* CentroVision^®^ L Forte

**Fig. 4:**
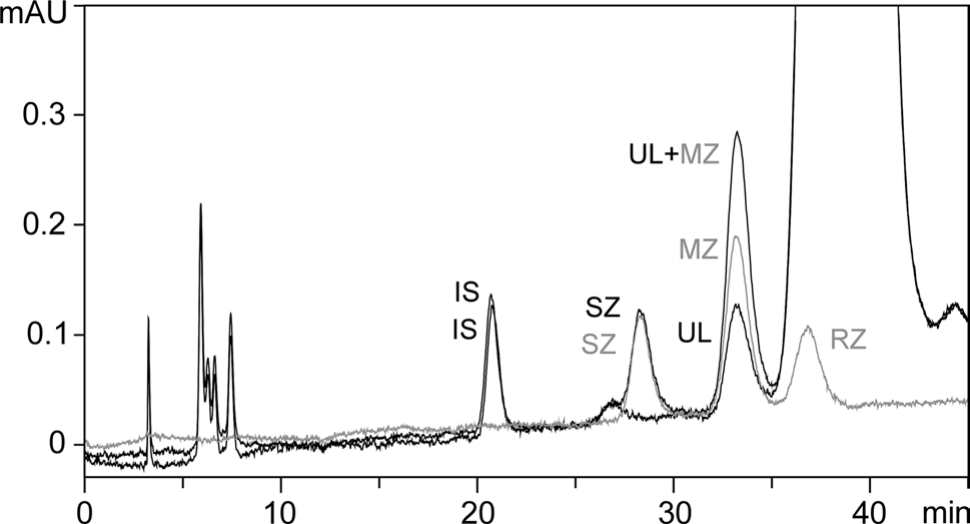
Spiking of the putative *meso*-zeaxanthin peak of Ultra Lutein^®^ with standard *meso*-zeaxanthin. In *grey*, HPLC profile of standard zeaxanthin enantiomeric mixture. UL, putative *meso*-zeaxanthin peak in Ultra Lutein^®^; UL + MZ, *meso*-zeaxanthin putative peak of Ultra Lutein^®^ spiked with standard zeaxanthin enantiomeric mixture. *IS* internal standard (cantaxanthin)

As shown in Fig. 3, we report that in six of seven products not declaring MZ, we found a peak on the HPLC chromatogram with the same spectrophotometric characteristics of MZ. Only for Preservision AREDS2^®^, we cannot assert whether MZ is present (or indeed absent) in this formulation, due to the quality of the spectra obtained from the putative MZ peak found in this supplement (Fig. 3c, peak II). The identity of the putative MZ peaks detected was confirmed by a spiking experiment performed with one of the formulations (Ultra Lutein^®^) using the MZ standard (Fig. 4). MZ concentration was quantified for these supplements (Table 1).

**Fig. 5:**
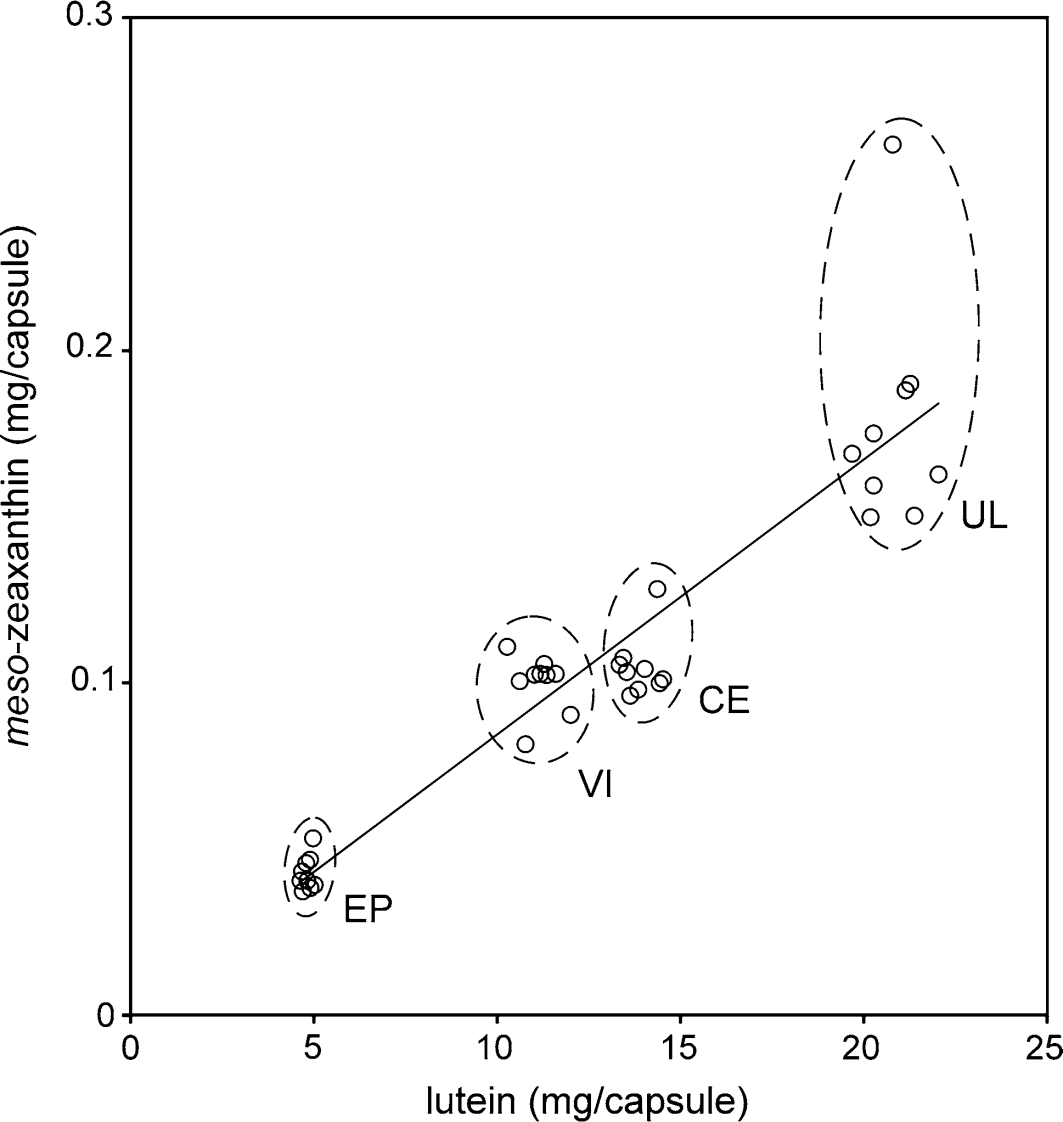
Lutein and *meso*-zeaxanthin concentrations measured in a selection of supplements sourcing FloraGLO^®^ Lutein and least-squares line of best fit. Each *circle* represents the lutein and *meso*-zeaxanthin concentration measured in a single capsule; nine single measurements from three different batches are represented for each formulation. *EP* Eyepromise Restore^®^, *VI* Vitalux Plus^®^, *CE* CentroVision^®^ L Forte, *UL* Ultra Lutein^®^

Five of these six products used the same L source (FloraGLO^®^ Lutein), suggesting that this carotenoid is present in this L source. Regression analysis, to test the relationship between detected MZ and L (using the four products containing MZ below 1 % of total carotenoid content), revealed a strong positive and statistically significant relationship, suggesting that the amount of undeclared MZ is related to the concentration of L in these supplements (see Fig. 5, regression formula MZ = 0.0083L + 0.0017; *r*^2^ = 0.86; *P* < 0.001).

## Discussion

In this study, we tested the concordance of L, Z and MZ concentrations to product label claim. We found that most of the formulations contained L concentrations higher than declared, with the exception of Nutrof^®^omega and CentroVision^®^ L forte, but it should be noted that even for these two formulations, the 95 % confidence intervals did include the declared concentration [Nutrof^®^omega, 95.4 % (90.2–100.5 %); CentroVision^®^ L forte, 99.3 % (96.8–100.8 %), see Table 1]. Of note, the highest concentration of L detected in any of the formulations tested was only 121 % of declared content for three of them, suggesting that the concentrations of L are tightly and sparingly controlled by manufacturers. Thus, we report that all nine supplements achieved close to or slightly above-declared L concentrations.

With respect to MZ, the two formulations declaring this carotenoid (Macuhealth with LMZ3^®^ and Macushield^®^) achieved their target MZ concentrations. Indeed, even in the batch of Macuhealth with LMZ3^®^ containing the lowest concentrations of total carotenoids (97.5 %), MZ content was 104 % of declared content.

With respect to Z, Macuhealth with LMZ3^®^ and Macushield^®^ contained Z concentrations higher than declared, whereas Ocuvite^®^ L Plus, whose carotenoid source is not known, did not contain the concentration of Z declared on their product label (with only 60 % of the declared concentration detected in our experiment). In the formulations using FloraGLO^®^ Lutein, two formulations failed to achieve their Z target by substantial amounts (VitaluxPlus^®^ and Nutrof^®^omega, showing Z concentrations of 47 and 52 % of declared content, respectively). However, the rest of the formulations using FloraGLO^®^ Lutein contained concentrations of Z much higher than the declared content, ranging from 161 % in CentroVision^®^ L forte to 248 % in Ultra Lutein^®^. These disparities in Z content (47–248 % of declared content) are somewhat unexpected, given that the concentrations of L in these supplements did not diverge much (95–121 %) from their declared concentrations, and both of these carotenoids were provided by FloraGLO^®^ Lutein (i.e. FloraGLO^®^ Lutein was the source of L and Z for these products).

Also, we calculated the L:Z ratio for each product and compared it to the respective declared L:Z ratio. As given in Table 2, the 95 % confidence intervals for the L:Z ratio indicate that three supplements (Ultra Lutein^®^, CentroVision^®^ L forte and Preservision AREDS2^®^) are significantly below their target ratio, and three supplements (VitaluxPlus^®^, Ocuvite^®^ L Plus and Nutrof^®^omega) are significantly above their target ratio for L:Z. The origins of this over- or under-achievement are evident from the final column of Table 1. For example, for Ultra Lutein^®^, L is 104 % of target claim, whereas Z is 248 % of target claim, and the L:Z ratio is therefore much lower than declared. Accordingly, it appears that the reason for the observed discordance between declared and measured L:Z ratio in these products is mistaken declaration of Z concentration in these formulations. Of note, the 95 % confidence intervals for the L:Z ratio indicate that Macuhealth with LMZ3^®^ and Macushield^®^ achieved their declared ratio. It is important to point out that both of these products obtain their carotenoid blend from the same company (Industrial Orgánica S.A.). With respect to the L:MZ ratio for these formulations, they are marginally below target, and it appears that the reason for this finding is that the manufacturers declare the same concentration (10 mg per capsule) for each of these carotenoids, but the measured MZ concentrations are higher than the measured L concentrations in both products.

One of the most interesting and important findings from our study was the detection of MZ in products not declaring this carotenoid on the product label. Importantly, where MZ was detected, it was present in each of the three batches analysed for each formulation. Further, because we avoided saponification in our process, the observed MZ cannot be attributed to artifactual generation from L by our extraction method. To our knowledge, this is the first study to identify and quantify undeclared MZ in eye care formulations containing the macular carotenoids. The finding of MZ in five out of six products using FloraGLO^®^ Lutein and the strong correlation between measured L and MZ in four of these supplements suggests that this carotenoid is present in this source of L. Indeed, based upon the regression formula obtained, a supplement containing 10 mg of L per capsule from FloraGLO^®^ Lutein would contain circa 0.085 mg of MZ.

The presence of small amounts of undeclared MZ in L sources (below 1 % in FloraGLO^®^ Lutein) is likely unavoidable due to the saponification process performed to generate L. However, these low concentrations of MZ reported should not be dismissed, from either a clinical or research perspective, as it has been shown that MZ was detected in the serum of subjects after supplementation with Ultra^®^ Lutein daily for 4 weeks [30]. We believe that it is important that MZ is declared on the product label (even if it is present in small concentrations) of formulations that do indeed contain this carotenoid.

Of interest, the formulation Nutrof^®^omega contains MZ concentrations higher than expected for a supplement using FloraGLO^®^ Lutein, and a similar result is exhibited by Ocuvite^®^ L Plus, whose carotenoid source is not known. We reported that these two formulations, together with VitaluxPlus^®^, contained only circa 50 % of declared Z. We hypothesize that the manufacturers of Ocuvite^®^ L Plus and Nutrof^®^omega added (covertly or inadvertently) MZ in an attempt to achieve the declared Z content and L:Z ratio; however, we have no direct evidence to support this statement. This practice has been previously reported [29] where it was suggested that this was performed because MZ is less expensive than Z.

## Conclusion

In conclusion, each commercially available formulation tested achieved a minimum of declared L content, but this was not the case for Z, with a high degree of variation between formulations. Six of seven formulations with undeclared MZ contained this carotenoid across several batches of product tested, probably as a result of using FloraGLO^®^ Lutein in at least four of them. This indicates that a greater degree of regulation is required for the use of commercially available supplements containing the macular carotenoids, and greater transparency by producers with respect to the source of the respective carotenoids is advised.
